# Gut microbiome dysregulation in noninfectious uveitis

**DOI:** 10.3389/fimmu.2025.1614304

**Published:** 2025-07-29

**Authors:** Mingzhu Liu, Jiawei Geng, Tao Liu, Xiaoli Liu

**Affiliations:** Ophthalmologic Center of the Second Hospital, Jilin University, Changchun, China

**Keywords:** noninfectious uveitis, gut microbiome, gut-eye axis, dysregulation, T regulatory cells, T helper 1/17 cell, treatment intervention

## Abstract

Noninfectious uveitis (NIU) is a vision-threatening autoimmune disease of the eye, but its pathogenesis is still not fully understood. Recently, accumulating evidence suggests that gut microbiome dysbiosis may affect the development and progression of NIU through potential mechanisms, including translocation, molecular mimicry, and bystander activation. Understanding the mechanisms of gut microbiome-host interactions, especially the gut-eye axis regulation, can offer a theoretical foundation for developing novel therapeutic strategies. We summarized current evidence on the dysregulation of gut microbiome and metabolites in NIU, and explored potential mechanisms involved. Furthermore, possible therapeutic measures are discussed, including probiotics, prebiotics, dietary modifications, antibiotic interventions, as well as fecal microbial transplantation, aiming to exert beneficial effects on NIU progression by reshaping the gut microbial composition.

## 1 Introduction

Uveitis is a series of intraocular inflammatory diseases involving the iris, ciliary body, choroid and adjacent structures (including the retina and optic nerve). It is one of the common causes of blindness, accounting for 15% of global vision impairment cases and 25% of blindness cases in developing countries (1, 2). Noninfectious uveitis (NIU) is presumed to be autoimmune-mediated uveitis. While a subset of cases is associated with systemic autoimmune diseases, such as Behcet’s disease (BD) and Vogt-Koyanagi-Harada syndrome (VKH), the majority remain idiopathic (3, 4).

**Figure 1:**
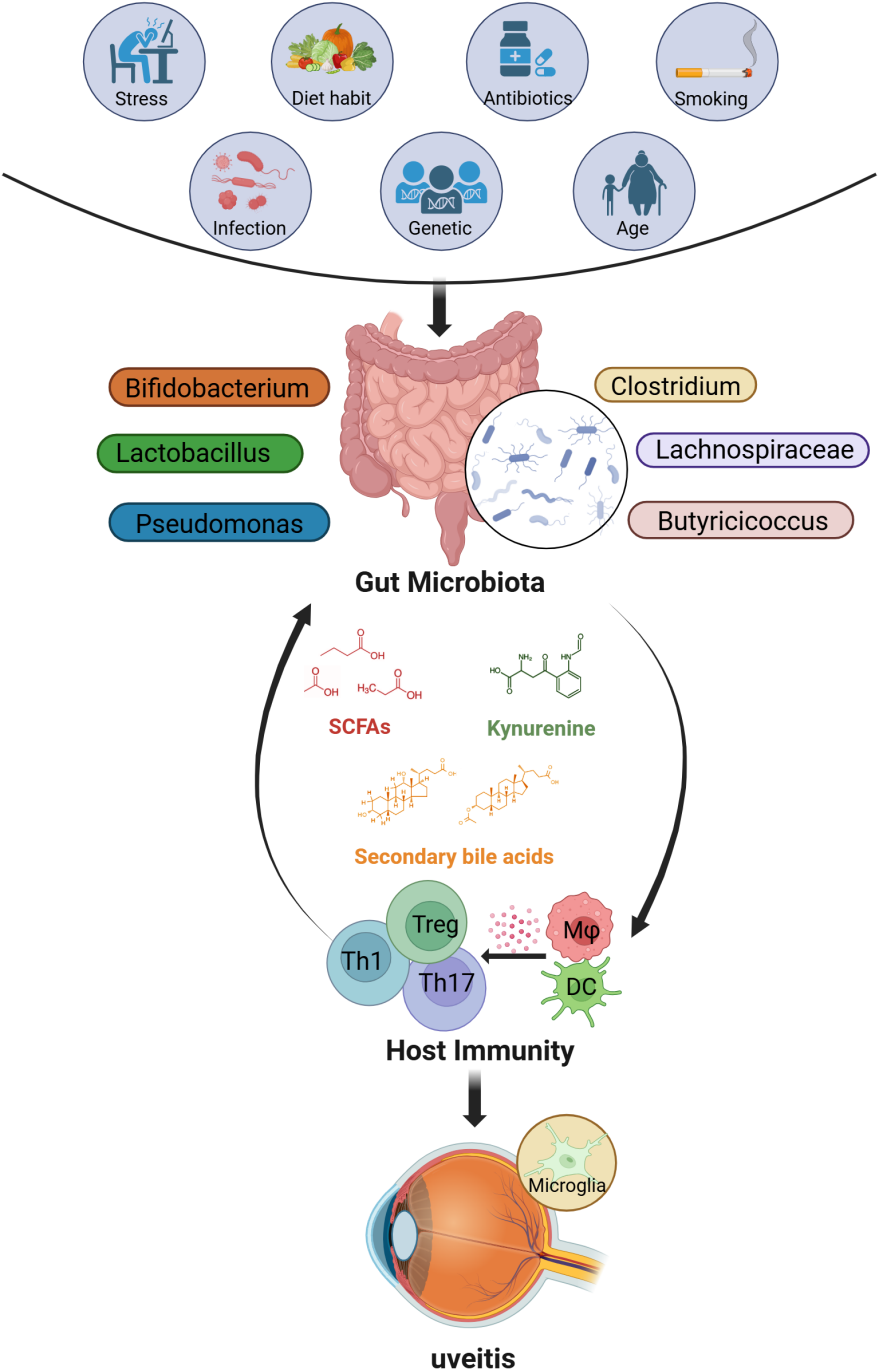
The factors that influence the gut microbiome and possible interactions between the gut and eye in uveitis. Many factors (such as stress, diet, antibiotics, smoking, infections, genetics, and age) may alter the composition of the gut microbiome. In most cases, these factors lead to changes in the abundance of SCFAs-producing bacteria (such as Clostridium, Lachnospiraceae, Butyricicoccus), lactic acid-producing bacteria (such as Bifidobacterium, Lactobacillus), and opportunistic pathogens like Pseudomonas, thereby affecting the levels of related metabolites (including SCFAs, secondary bile acids, and tryptophan metabolites such as kynurenine). Ultimately, an imbalance in the gut microbiome may regulate host immunity and drive the development of uveitis. (Created with BioRender.com) SCFAs, short-chain fatty acids; Th, T helper cells; Treg, regulatory T cells; Mφ, macrophage; DC, Dendritic cells.

The pathogenesis of NIU is thought to involve both genetic and environmental factors. Among genetic predispositions, specific human leukocyte antigen (HLA) genes are strongly implicated, such as HLA-B51 in BD (5), HLA-DR4/DR53 in VKH (6), and HLA-B27 in acute anterior uveitis (AAU) (7). As for environmental factors, increasing evidence has found a critical role for the gut microbiome in modulating immune responses relevant to NIU. However, it can also be modulated by multiple factors, including smoking, diet habits, drugs, age, and psychological stress (**Figure 1**) (8, 9). The gut microbiome is a complex microecosystem comprising bacteria, viruses, fungi, archaea, and thousands of unique metabolites that collectively shape local and systemic immune responses. Emerging evidence suggests that dysbiosis of the gut microbiome may be associated with disruptions in the balance between pro-inflammatory Th1/Th17 cells and Treg cells (10, 11), which is a key immunological hallmark of NIU. However, whether gut microbiome dysbiosis acts as a trigger and modulator of autoimmune responses in the context of NIU, or is a consequence of inflammation-driven immune imbalance and metabolic alterations during disease progression, remains a subject of debate.

Based on current evidence, we hypothesize that dysbiosis of the gut microbiome and its metabolites trigger gut inflammation and increase intestinal permeability (“leaky gut”). This allows the microbiome and its components to translocate into the lamina propria and systemic circulation, promoting aberrant immune activation. Moreover, gut microbiome and metabolites that have entered the lamina propria may further induce cross-reactive T cell responses or amplify the immune activation state through mechanisms such as molecular mimicry and bystander activation, thereby contributing to the initiation and progression of NIU. In this review, we discuss the alterations of gut microbiome and its metabolites in NIU and outline the potential mechanisms linking these changes to the disease. Finally, we propose future directions for microbiota-targeted interventions as emerging therapeutic strategies for NIU.

## 2 Gut microbiome dysbiosis in NIU

### 2.1 Dysbiosis of gut bacteriome

Multiple studies have shown gut microbiome dysbiosis in patients with NIU (**Table 1**). Although the composition of gut microbiome is influenced by various factors, such as the study population, sample collection, detecting techniques, and geographic region, representative patterns of dysbiosis can still be consistently identified. These patterns primarily include (1) changes in microbial diversity and (2) compositional shifts in specific bacterial taxa.

**Table 1:** The alteration of gut microbiome in uveitis.

Uveitis type and participants	Altered abundance in uveitis	Country
BD		
22 BD vs 16 co-habiting HCs (15)	**↓: Genus:** Roseburia, Subdoligranulum (belong to the Clostridium clusters XIVa and IV)	Italy (2015)
12 BD vs 12 HCs (25)	**↑: Genus:** Bifidobacterium, Eggerthella, Lactobacillus. **↓: Genus:** Megamonas, Prevotella	Japan (2016)
24 untreated, active BD vs 52 HCs (16)	**↑: Genus:** sulfate-reducing bacteria, opportunistic pathogens. **↓: Genus:** butyrate-producing bacteria, methanogens	China (2018)
15 untreated, active BD vs 43 HCs (16)	**↑: Genus:** Bifidobacterium, Prevotella, Scardovia	
13 BD vs 27 HCs (19)	**↑: Genus:** Acidaminococcus; **Species:** Eggerthella lenta, Lactobacillus mucosae, Bifidobacterium bifidum, Lactobacillus iners, Streptococcus species, Lactobacillus salivarius. **↓: Genus:** Butyrivibrio, Filifactor; **Species:** Megamonas hypermegale, Streptococcus infantis	Japan (2019)
27 BD vs 10 HCs (32)	**↑: Genus:** Actinomyces, Libanicoccus, Collinsella, Eggerthella, Enetrohabdus, Catenibacterium, and Enterobacter. **↓: Genus:** Bacteroides, Cricetibacter, Alistipes, Lachnospira, Dielma, Akkermansia, Sutterella, Anaerofilum, Ruminococcease-UCG007, Acetanaerobacterium, and Copropaacter.	Turkey (2020)
7 BD vs 16 HCs (148)	**↑: Family:** Succinivibrionaceae, Veillonellaceae; **Genus:** Succinivibrio, Mitsuokella. **↓: Family:** Bacterioidaceae	Turkey (2020)
Dutch cohort: 19 BD vs 17 HCs Italy cohort: 13 BD vs 15 HCs (18)	**↑:** IgA-coating of Bifidobacterium spp., Dorea spp. and Ruminococcus bromii species. **↓: Genus:** unclassified Barnesiellaceae, Lachnospira; IgA-coated Erysipelotrichaceae spp. and RF38 spp	Italy (2020)
Italy cohort: 18 BD vs 15 HCs (18)	**↑: Family:** Spirochaetaceae, Dethiosulfovibrionaceae; **Genus:** Treponema, TG5	
9 BD vs 9 Healthy relatives (12)	**↑: Genus:** Lachnoanaerobaculum; **Species:** Bacteroides uniformis	Korea (2021)
23 BD vs 12 HCs (149)	**↓: Genus:** Bacteroides, Acinetobacter, and Subdoligranulum; **Species:** Bacteroides fragilis, SCFAs-producing bacteria	Korea (2024)
11 BD vs 18 HCs and 15 Fuchs (87)	**↑: Genus:** Peptostreptococcaceae_unclassified, Eikenella. **↓: Genus:** Fusicatenibacter, *Stenotrophomona*	China (2024)
VKH		
55 untreated, active VKH vs HCs (17)	**↑: Genus:** Gram-negative bacteria. **↓: Genus:** butyrate-producing bacteria, lactate-producing bacteria, methanogens	China (2020)
11 untreated, active VKH vs HCs (24)	**↑: Genus:** Stomatoculum. Pseudomonas, Lachnoanaerobaculum. **↓: Genus:** Gordonibacter, Slackia	China (2022)
16 untreated, active VKH vs HCs (29)	**↑: Genus:** Pseudomonas, Exigubacterium, Pediococcus, Ligilactobacillus, Acinetobacter, Herbasperillum and HT002. **↓: Genus:** Lawsonia, Photobacterium, Rhodococcus, Gardnerella, Lactobacillus and Butyricicoccus	China (2023)
AU		
11 AAU vs 18 HCs (29)	**↑: Genus:** Liquorilactobacillus, Lentilactobacillus, Cetobacterium, Absiella, Actinobacillus, Lachnospiraceae_UCG-010, Eubacterium_brachy_group and Mitochondria_unclassified. **↓: Genus:** Helicobacter, Limosilactobacillus and Candisatus_Arthromitus	China (2023)
20 HLA B27+ AU vs 22 HLA B27- HCs (150)	**HLA+ AU vs HLA- HCs: ↓:** Eubacterium ramulus; **HLA+ AU vs HLA+ HCs: ↓:** Bacteroides caccae; **HLA+ active AU vs HLA- HCs:↑:** Phocaeicola vulgatus; **↓:**Eubacterium ramulus	Switzerland (2024)
15 Fuchs vs 18 HCs and 11 BD (87)	**↑: Genus:** Filifactor, Frisingicoccus, Bacteroidales_unclassified, Oscillospira, Delftia. **↓: Genus:** Prevotellaceae_NK3B31_group, Eubacterium_nodatum_group, Pantoea	China (2024)
Others		
13 UVT vs 13 HCs (151)	**↑: Genus:** Prevotella (proinflammatory) and Streptococcus (pathogenic). **↓: Genus:** Faecalibacterium, Bacteroides, Lachnospira, Ruminococcus	India (2018)
Animal models		
B10.RIII EAU mice (14)	3 weeks post-immunization: **↑: Genus:** Clostridium, Coprococcus, Dorea, Adlecreutzia, Desulfovibrio, Lactobacillus. **↓: Genus:** Ruminococcus, Oscillospora, Turicibacter, and Anaeroplasma	United States (2016)
B10.RIII EAU mice (13)	2 weeks post-immunization: **↑: Genus:** Anaerostipes. **↓: Genus:** Ruminococcus, Desulfovibrio	United States (2019)
C57BL/6J EAU mice (73)	**↑: Family:** Prevotellaceae. **↓: Family:** Ruminococcaceae, Lachnospiraceae, and Eggerthellaceae	China (2021)

Behcet’s disease (BD); Vogt-Koyanagi-Harada (VKH) disease; anterior uveitis (AU); healthy controls (HCs); Uveitis (UVT); Experimental autoimmune uveitis (EAU). BD, VKH, anterior uveitis, and others are uveitis subtypes. Species, Genus, and Family indicate taxonomic levels. ↑ = increased in disease; ↓ = decreased. HLA⁺ AU vs HLA⁻ HCs: HLA-positive anterior uveitis vs HLA-negative healthy controls. HLA⁺ anterior uveitis vs HLA⁺ HCs: HLA-positive anterior uveitis vs HLA-positive healthy controls. HLA⁺ active AU vs HLA⁻ HCs: active HLA-positive anterior uveitis vs HLA-negative healthy controls.

#### 2.1.1 Changes in microbial diversity

Most studies reported that the overall community structure of patients with uveitis differed from that of healthy controls, with significant changes in both α and β diversity. A longitudinal analysis of BD patients showed that during transition from active to inactive disease stages, α diversity dropped significantly (12). Similar diversity fluctuations have been observed in experimental autoimmune uveitis (EAU) models, indicating dynamic microbial instability during disease progression (13, 14).

#### 2.1.2 Compositional shifts in specific bacterial taxa

A prominent feature of composition dysbiosis in NIU is the reduction of beneficial SCFA-producing bacteria, including *Clostridium* clusters XIVa and IV (15–17), *Lachnospiraceae* (18), and *Butyricicoccus* (19). These bacteria possess anti-inflammatory properties, and their decreased abundance may reduce intestinal SCFA levels, which triggers Th17/Treg immune imbalance and disrupts the intestinal barrier function, affecting immune homeostasis (20, 21).

Another characteristic of composition dysbiosis involves shifts in lactic acid-producing bacteria, which vary across NIU subtypes. Many strains of lactic acid-producing bacteria, such as *Bifidobacterium* and *Lactobacillus*, are widely used to maintain gut homeostasis and modulate immune responses (22, 23). In patients with active VKH, the abundance of *Bifidobacterium* and *Lactobacillus* is significantly reduced (17, 24), which may impair intestinal barrier function and exacerbate systemic inflammation. However, in BD patients, *Bifidobacterium* and *Lactobacillus* are significantly increased (19, 25), and this change may influence the intestinal microenvironment through multiple mechanisms. On one hand, excessive production of lactic acid may lower the intestinal pH, inhibiting the survival of certain commensal bacteria and thereby disrupting gut microbiome balance (26). On the other hand, the abundance of sulfate-reducing bacteria (SRB) is also significantly increased in BD patients (16). SRB competes with butyrate-producing bacteria for substrates, utilizing lactic acid to generate the cytotoxic byproduct hydrogen sulfide, such as H_2_S, which exacerbates pro-inflammatory responses and impairs intestinal epithelial barrier function (27, 28). Furthermore, different bacterial strains may exhibit distinct metabolic profiles and immune mechanisms. These differences suggest that different types of uveitis may involve distinct patterns and mechanisms of intestinal microbiome imbalance, and provide potential directions for future personalized therapeutic strategies targeting intestinal flora.

-Overgrowth of opportunistic pathogens is another characteristic pattern of taxonomic dysbiosis in NIU. Our research group consistently observed a significant enrichment of *Pseudomonas* in VKH patients across two independent cohorts (24, 29). Further analysis revealed that the abundance of *Pseudomonas* was significantly higher in VKH patients compared to those with noninfectious scleritis or AAU, suggesting its potential critical role in the onset or progression of VKH. *Pseudomonas* can produce lipopolysaccharide (LPS) and peptidoglycan (PGN), which act on Toll-like receptor 4 (TLR4) and NOD-like receptors (NLRs), thereby activating host immune responses (30). Additionally, we also found that the abundance of *Pseudomonas* was significantly negatively correlated with biotin (vitamin B7) levels (29). Long-term biotin deficiency can lead to symptoms such as alopecia and poliosis, which are hallmark clinical manifestations of VKH patients (31).

In addition to baseline dysbiosis, disease stage and treatment status also influence gut microbiota taxonomic composition. By comparing the gut microbiome of VKH patients before and after immunosuppressive treatment (with active intraocular inflammation before treatment and without intraocular inflammation after treatment), Zi et al. found that composition dysbiosis partially recovered after treatment, in parallel with the resolution of intraocular inflammation. The primary bacterial alterations included a decreased abundance of *Acidaminococcus* sp. BV3L6 (positively correlated with VKH), alongside increased abundance of *Proteobacteria* bacterium CAG495, *Azospirillum* sp. CAG260, and *Alistipes* sp. CAG435 (negatively correlated with VKH) (17). Meanwhile, they also observed that *Bacteroides*. spp, *Prevotella*. spp, *Paraburkholdria*. spp and *Listeria*. spp were associated with recurrence after treatment (17). Parallel findings emerged in EAU. In B10.RIII mice immunized with interphotoreceptor retinoid-binding protein 161-180 (IRBP161-180), gut microbiota composition was analyzed on day 7 (pre-onset), day 14 (peak onset), and day 21 (chronic phase). The differences in bacterial composition became more pronounced as the disease progressed. Notably, *Desulfovibrio* was enriched in the non-immunized group during the peak phase, while it was enriched in the immunized group at the chronic phase (13, 14). Subgroup analyses based on clinical phenotypes in BD also revealed distinctive microbial signatures. The skin mucosal group was characterized by *Dialister*, *Intestinomonas*, and *Marvinbryantia*, the vasculitis group by *Gemella*, and the uveitis group by *Lachnospiraceae* NK4A136 (32). Another study also found that in patients with active BD, the abundance of *Bifidobacterium adolescentis* was higher in those with uveitis compared to those without uveitis (12). These findings suggest that while certain dysbiosis patterns are shared among NIU patients, disease activity, treatment, and clinical subtype are important modifiers of gut microbial structure, which may play distinct roles in different inflammatory mechanisms.

### 2.2 Dysbiosis of gut mycobiome

In healthy individuals, fungi constitute only a minor component of the gastrointestinal microbiome, representing approximately 0.01% to 0.1% of metagenomic reads in fecal samples (33). Compared to the bacterial microbiome, the gut mycobiota displays markedly lower diversity and is predominantly composed of *Saccharomyces*, *Malassezia*, and *Candida* (34). Compared to healthy controls, nine fungal genera were found to be significantly enriched in patients with uveitis. Among them, *Aspergillus gracilis*, *Candida glabrata*, *Malassezia globosa*, *M. restricta*, and *Issatchenkia* sp. AUMC 7766 are known opportunistic pathogens (35). Furthermore, gut microbiome interaction network analysis revealed multiple positive or negative correlations between fungal and bacterial taxa in uveitis patients (35), indicating that fungi may contribute to microbial dysbiosis. Similarly, in the EAU mouse model, antibiotic-induced bacterial depletion was accompanied by an overgrowth of gut fungi. This dynamic shift may be associated with reduced bacterial competition, ecological niche vacancy, and changes in host immune responses (36).

### 2.3 Dysbiosis of gut virome

The human virome is predominantly composed of eukaryotic viruses (viruses that target human cells) and prokaryotic viruses (viruses that infect bacteria and archaea). Eukaryotic viruses account for less than 10% of the virome and primarily include herpesviruses, anelloviruses, and adenoviruses. In most cases, these viruses remain in a latent or dormant state and may contribute to the initiation of immune responses (37). Bacteriophages (also known as bacterial viruses) far exceed the proportion of eukaryotic viruses, accounting for over 90% (37). With advances in high-throughput sequencing technologies (such as metagenomic sequencing) and bioinformatics analyses, numerous studies have revealed the complex relationships between the gut virome—particularly bacteriophages—and human diseases, including metabolic (38, 39) and autoimmune disorders (40, 41). The dominant phages found in the gut include *Caudovirales* (dsDNA viruses), *Microviridae* (ssDNA viruses), and cross-assembly phages (crAssphages) (42). These phages may influence human health either by modulating the bacterial microbiome or through direct interactions with the host immune system.

Viral infection may serve as a potential trigger for uveitis. Epstein-Barr virus (EBV) DNA was detected by polymerase chain reaction (PCR) in the vitreous fluid and cerebrospinal fluid of VKH patients (43, 44). In VKH disease, tyrosinase and gp100 are key target antigens involved in the pathogenic immune response. Database screening revealed that the tyrosinase peptide 450–462 shared a homologous amino acid sequence with the cytomegalovirus envelope glycoprotein H peptide (CMV-egH290-302), and further studies confirmed cross-reactive T cell responses between the two peptides (45, 46). Similarly, preliminary evidence suggests that BD may also be associated with viral infections, including EBV, CMV, herpes simplex virus type 1 (HSV-1), varicella-zoster virus (VZV) (47). Early studies have reported the detection of HSV-1 DNA in peripheral blood leukocytes, saliva, and oral ulcer samples of BD patients (48, 49). To date, the most widely accepted theory regarding infectious triggers of BD is that certain microbial antigens share high sequence homology with human proteins, thereby inducing cross-reactive immune responses (50). However, no studies have yet specifically examined the role of the gut virome in uveitis, and its potential role remains to be elucidated.

## 3 Gut metabolite disturbances in NIU

**Figure 2:**
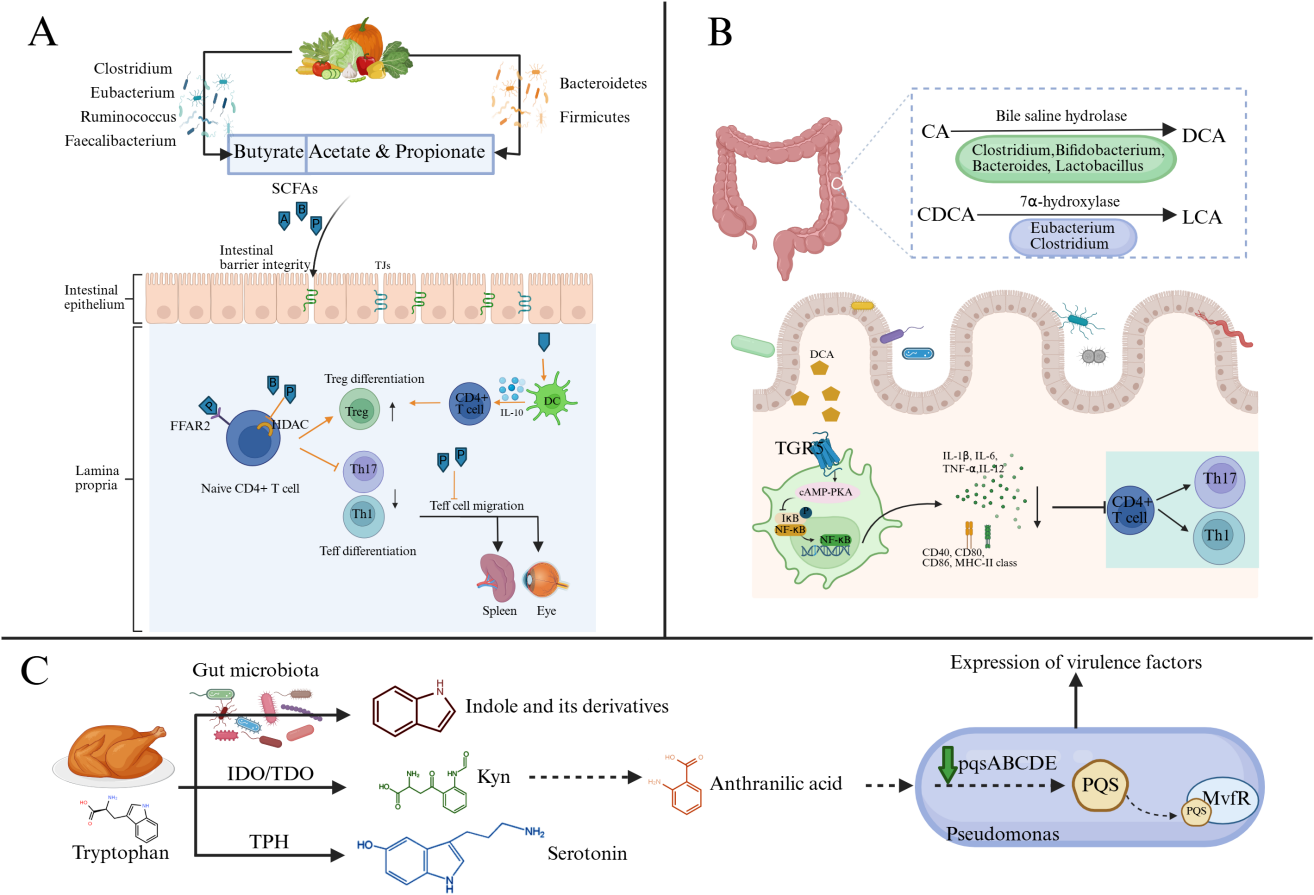
Major microbial metabolic hypothesized pathways to impact uveitis risk and severity. **(A)** The gut microbiome breaks down host dietary fibers and indigestible carbohydrates to produce SCFAs. SCFAs can maintain the integrity of the intestinal barrier in uveitis and regulate immune cells by binding to FFAR2 or inhibiting HDAC. In addition, propionate can inhibit the migration of Teff cells, especially Th1 cells, from the gut to extra-intestinal lymph nodes. **(B)** CA and CDCA are metabolized by the gut microbiome to produce DCA and LCA. These two secondary bile acids can bind to TGR5, activate the cAMP-PKA signaling pathway, and inhibit NF-κB-mediated DC activation, thereby reducing the expression of proinflammatory cytokines and costimulatory molecules. **(C)** The tryptophan metabolism pathway includes the indole pathway, kynurenine pathway, and serotonin pathway. The indole pathway occurs in the intestinal lumen via the gut microbiome. Kynurenine can be further metabolized into anthranilic acid, which is a precursor of the quorum-sensing signal molecule PQS. PQS is synthesized by Pseudomonas through the pqsABCDE gene cluster. Upon binding with MvfR, it regulates the quorum-sensing system of Pseudomonas, thereby influencing the expression of its virulence factors. (Created with BioRender.com) SCFAs, short-chain fatty acids (A, acetate; P, propionate; B, butyrate); Th, T helper cells; Treg, regulatory T cells; DC, Dendritic cells; FFAR2, Free Fatty Acid Receptor 2; HDAC, Histone Deacetylase; CA, cholic acid; CDCA, chenodeoxycholic acid; DCA, deoxycholic acid; LCA, lithocholic acid; TGR5, T G-protein coupled receptor 5; IDO, indoleamine 2,3-dioxygenase; TDO, tryptophan 2,3-dioxygenase; TPH, tryptophan hydroxylase; Kyn, kynurenine; PQS, Pseudomonas Quinolone Signal; MvfR, Multiple Virulence Factor Regulator.

Clinical and experimental evidence indicates that alterations in the gut microbiome composition in patients with uveitis can lead to disturbances in metabolite profiles (**Table 2**). These metabolic changes were manifested in the metabolism of carbohydrates, fatty acids, amino acids, and bile acids (DAs). Here, we will focus on the changes of several key metabolites and their potential implications (**Figure 2**).

**Table 2:** The alteration of metabolites related to gut microbiome in uveitis.

Disease	Samples	Analytical techniques	Increased metabolites	Decreased metabolites	Country
BD					
22 BD vs 16 co-habiting HCs (15)	fecal samples	GC-MS	/	Fatty acid (butyrate)	Italy (2015)
35 BD vs 35 HCs (88)	serum	GC/TOF-MS	Fatty acid (decanoic acid) Carbohydrates (fructose, tagatose)	Fatty acid (linoleic acid, oleic acid)	Korea (2018)
24 BD vs 25 HCs (89)	serum	UPLC-QTOF-MS^E^	Fatty acid (linoleic acid, arachidonic acid, oleic acid)	Phospholipid (phosphatidylcholines)	China (2018)
15 BD vs 15 senile cataract controls (152)	aqueous humor	LC/MS	Fatty acid (palmitic acid and oleic acid); Amino acid (L-phenylalanine, D-arginine, L-alanine, arginine-cysteine, L-arginine et al.)	Amino acid (N-acetyl-L-aspartate, et al.)	China (2021)
10 BD vs 10 HCs (153)	plasma	LC/MS	Lipid metabolites (triglyceride, sphingomyelin)	Lipid metabolites (ceramides, diacylglycerol, free fatty acid)	China (2022)
120 BD vs 120 HCs (82)	serum	HPLC–MS/MS	kynurenine, kynurenic acid, 3-hydroxyanthranilic acid, 3-hydroxykynurenine, quinolinic acid, Kynurenine/tryptophan	tryptophan	Turkey (2022)
40 BD vs 18 HCs (91)	plasma and PBMC	GC-MS and LC/MS	Fatty acid (linoleic acid); Phospholipid (lysophosphatidylcholine, sphingosine 1-phosphate and ether-linked lysophosphatidylethanolamine)	Carbohydrates (glucose, fructose, tagatose, et al.)	Korea (2023)
11 BD vs 18 HCs (87)	fecal samples	LC–MS/MS	Fatty acid (palmitic acid, myristic acid); Vitamins (ascorbic acid) Serotonin	Vitamins (delta-tocopherol)	China (2024)
30 BD vs 20 HCs (90)	plasma	LC-MS	quinate, stearidonic acid, capric acid		China (2025)
12 active BD vs 18 inactive BD (90)			1-methyladenosine, genipin, methylmalonic acid, ascorbate		
VKH					
28 active VKH vs 30 HCs (154)	plasma	LC-MS/MS	Carbohydrates (D-mannose); Amino acid (L-lysine)	Fatty acid (stearic acid)	China (2020)
27 inactive VKH vs HCs (154)			stearic acid		
18 active VKH vs 18 inactive VKH and 22 HCs (81)	plasma	LC-MS	Amino acid (Kynurenine/tryptophan)		China (2020)
15 VKH vs 15 senile cataract controls (152)	aqueous humor	LC/MS	Fatty acid (palmitic acid and oleic acid); Amino acid (ornithine et al.)	Amino acid (L-histidine, L-valine et al.)	China (2021)
16 VKH vs 18 HCs (29)	fecal samples	LC–MS/MS		Vitamins (biotin, downstream metabolites of niacin: 1-methylnicotinamide and N1-methyl-4-pyridone3-carbaxamide)	China (2023)
AU					
38 AAU vs 40 HCs (74)	fecal samples	GC-MS	6-deoxy-D-glucose 1, linoleic acid, N-Acetyl-beta-D-mannosamine 3, shikimic acid, azelaic acid, Isomaltose 1, and palmitoleic acid		China (2018)
11 AAU vs 18 HCs (29)	fecal samples	LC–MS/MS		6-Keto-prostaglandin F1a	China (2023)
20 Fuchs vs 20 senile cataract controls (155)	aqueous humor	LC/MS	Amino acid (DL-serine et al.)		China (2022)
15 Fuchs vs 18 HCs (87)	fecal samples	LC–MS/MS	Amino acid (D/L-serine, L-threonine); Fatty acid (linoleic acid)		China (2024)

Behcet’s disease (BD); Vogt-Koyanagi-Harada (VKH) disease; anterior uveitis (AU); healthy controls (HCs); the gas chromatograph -mass spectrometry (GC–MS); the gas chromatography with time-of-flight mass spectrometry (GC/TOF-MS); the ultra-performance liquid chromatography-quadrupole time-of-flight mass spectrometry (UPLC-QTOF-MS^E^); liquid chromatography/mass spectrometry (LC/MS); high performance liquid chromatography–tandem mass spectrometry (HPLC–MS/MS); liquid chromatography-tandem mass spectrometry (LC-MS/MS).

### 3.1 The metabolism of short chain fatty acids

SCFAs, such as acetate (C2), propionate (C3), and butyrate (C4), are produced through the fermentation of dietary fibers and indigestible carbohydrates (such as resistant starch) by the gut microbiome (51). Different types of SCFAs are metabolized by different bacterial taxa. *Eubacterium*, *Ruminococcus*, *Faecalibacterium*, and *Clostridium* clusters IV and XIVa dominate the production of butyrate, while acetate and propionate are associated with *Bacteroidetes* and *Firmicutes* (52–55). The immunomodulatory effects of SCFAs have been comprehensively reviewed (51, 56). In addition to serving as energy sources for the host and gut microbes (57), the possible mechanisms include: 1) maintaining the integrity of the intestinal barrier (58); 2) improving the inflammatory environment by inhibiting the expression of pro-inflammatory cytokines (IL-8, IL-6, IL-1β and TNFα) and promoting the production of anti-inflammatory cytokines (IL-10, TGF-β) (59); 3) inducing tolerance and an anti-inflammatory phenotype in various immune cells (including neutrophils, macrophages, Foxp3+ Treg cells, B cells, and microglia) through G protein-coupled receptors (GPCRs, GPR41, GPR43, and GPR109a) or by inhibiting histone deacetylases (HDAC) (60–64).

Studies have found that, compared to healthy relatives, the levels of butyrate in fecal samples from BD patients were significantly reduced and positively correlated with *Roseburia* (15). Furthermore, the transplantation of fecal samples from patients with active BD into B10.RIII mice led to a reduction in the concentrations of propionate, butyrate, and valerate (65). Exogenous administration of SCFAs, particularly propionate and butyrate, significantly alleviated ocular inflammation in C57BL/6 EAU mice (66, 67). Nakamura et al. demonstrated using Kaede transgenic mice (expressing photoconvertible fluorescent Kaede protein) that propionate suppressed the migration of effector T cells (Teff) between intestinal and extraintestinal tissues. However, no protective effect of propionate was observed in B10.RIII EAU mice, which may involve multiple mechanisms, including mouse strain-specific gut microbiome composition. A high-pectin diet can increase the concentrations of acetate and propionate in the gut, exerting effects on EAU mice similar to propionate administration. Notably, pectin supplementation can upregulate the expression of the SCFA receptor FFAR2/GPR43 in the ileum during the peak phase of uveitis (68). In addition, the inhibition of Th17 differentiation mediated by butyrate may be achieved through the activation of nuclear factor erythroid 2-related factor 2 (Nrf2)/heme oxygenase 1 (HO-1) pathway and the suppression of IL-6 receptor (IL-6R) expression (67). In BD patients, a butyrate-rich diet was also observed to modulate blood redox status and enhance fibrinolysis. However, it did not significantly alter gut microbiome composition or SCFA production, which may require a longer period of nutritional intervention (69). The above evidence highlights the role of gut microbiome-induced changes in SCFAs in the immune regulation of NIU.

### 3.2 The metabolism of bile acids

Cholesterol is metabolized into two primary bile acids in the liver: cholic acid (CA) and chenodeoxycholic acid (CDCA) (70). After entering the intestine, CA and CDCA are metabolized to deoxycholic acid (DCA) and lithocholic acid (LCA) by microbial bile saline lyase (BSH, mainly expressed by *Clostridium*, *Bacteroides*, *Bifidobacterium*, and *Lactobacillus*) and 7α-dehydroxylase (mainly expressed by *Eubacterium* and *Clostridium*), respectively (70, 71). BAs play a key role in the host immune response through several GPCRs or nuclear receptors, such as farnesoid X receptor (FXR). Among them, CDCA was identified as the most effective FXR ligand, while DCA and LCA were the preferred agonists of Takeda G protein-coupled receptor 5 (TGR5) (70).

The expression of TGR5 in M1 macrophages of patients with active VKH is significantly lower than that of healthy controls (72). Further experiments showed that TGR5 activation could induce the transformation of M1 (inflammatory) to M2 (anti-inflammatory) macrophages and inhibit the differentiation of Th1 and Th17 cells (72). The same research group also found that the levels of secondary bile acids (DCA, LCA, etc.) were reduced in the feces and serum of C57BL/6 EAU mice, and the proportion of secondary bile acids was positively correlated with the level of *Clostridium scindens* in the feces (73). *C. scindens* can convert primary bile acids into secondary bile acids, and its levels were reduced in patients with BD and AAU (16, 74). Studies have shown that the colonization of *C. scindens* restored the composition of bile acids in the feces and reduced the severity of EAU (73). Additionally, a diet rich in DCA or LCA significantly alleviated ocular inflammation in EAU mice (73, 75). This protective effect is closely related to the activation of the TGR5 receptor mediated by DCA, which inhibits nuclear factor kB (NF-kB)-mediated DC activation via the cyclic AMP (cAMP)-protein kinase A (PKA) signaling pathway, thereby reducing inflammation levels (73). However, the specific mechanisms of bile acid metabolism in uveitis still require further validation.

### 3.3 The metabolism of tryptophan

Tryptophan (Trp) is an essential amino acid for the human body and can be metabolized by the gut microbiome into various indole-containing compounds. For example, *Escherichia coli*, *Clostridium*, and *Bacteroides* can produce indole through tryptophanase (76). These indole derivatives can act as effective immune regulators by binding to the aryl hydrocarbon receptor (AhR) (77). Additionally, tryptophan can also be metabolized via the kynurenine (Tyn) and serotonin pathways. *Pseudomonas* can utilize the kynurenine pathway to generate anthranilate, and then synthesize the quorum-sensing signaling molecule 2-heptyl-3-hydroxy-4-quinolone (Pseudomonas quinolone signal, PQS). Studies have shown that PQS regulated the immune response in arthritis by inhibiting the differentiation of CD4^+^ IFNγ^+^ cells (78).

The blood Kyn/Trp ratio is commonly used as a marker to evaluate the activity of indoleamine 2, 3-dioxygenase (IDO) in chronic immune activation (79). Previous studies have shown that the Kyn/Trp ratio and Kyn concentration in serum and urine of patients with active uveitis were significantly higher than those of healthy controls, and the Kyn/Trp ratio is even higher in patients with remission uveitis (80). Another study observed a similar trend in VKH patients, except that the Kpn/Trp ratio of VKH patients in the remission phase was similar to that of the control group (81). This may be due to the fact that the former study included multiple uveitis entities, while the latter only focused on VKH patients. *In vitro* experiments have shown that IDO can affect the antigen-presenting function by enhancing the expression of the cell surface marker CD86, whereas the downregulation of IDO may lead to a reduction in Tregs and an expansion of CD4+ T cells (81). Additionally, dysregulation of the kynurenine metabolism pathway has also been observed in BD patients, characterized by increased tryptophan degradation, along with elevated levels of kynurenine (KYN), kynurenic acid (KYNA), 3-hydroxyanthranilic acid (3HAA), 3-hydroxykynurenine (3HK), and quinolinic acid (QUIN) in serum. These metabolites have been implicated in BD disease activity, clinical manifestations, and inflammatory burden (82). Further research is needed to identify the key tryptophan metabolites involved in the immune response of different uveitis and their potential roles.

Although current studies have predominantly focused on the individual immunoregulatory functions of metabolites, emerging evidence indicates that these metabolites may interact through shared signaling pathways or have overlapping effects on immune homeostasis. For example, SCFAs could influence tryptophan metabolism by promoting the production of AhR ligands, which are known to exert anti-inflammatory effects and enhance mucosal barrier integrity (83). On the other hand, BAs regulate immune responses via FXR and TGR5 and may also modulate the gut bacteria composition involved in tryptophan degradation (84, 85). Furthermore, spore-forming bacteria, known producers of SCFAs, have also been implicated in stimulating serotonin biosynthesis from tryptophan by enhancing tryptophan hydroxylase (TPH)1 expression in colonic enterochromaffin cells (86). These findings suggest that SCFAs, BAs, and tryptophan metabolites may form an interlinked metabolic network that collectively shapes immune responses in the gut and potentially at distal sites such as the eye. However, the precise mechanisms underlying their crosstalk and relevance in NIU remain to be fully elucidated.

Additionally, our studies have highlighted the potential of gut microbiome-derived metabolites as non-invasive biomarkers in NIU. In BD, 19 fecal differential metabolites showed AUC values above 80% (87). Similarly, 31 metabolites distinguished Fuchs syndrome from controls with high accuracy, including serotonin (87). Moreover, two metabolites (–),-gallocatechin and vanillin, effectively differentiated VKH, while norfloxacin showed an AUC of 82% in distinguishing AAU from controls (29). Notably, other studies have also identified metabolite profiles from serum or plasma (88–91) and urine (92) as potential diagnostic biomarkers for BD. These findings suggest that microbiome-related metabolites may serve as accessible biomarkers for NIU diagnosis and stratification, although further validation in larger cohorts is needed.

## 4 The hypothesized mechanism of gut microbiome dysbiosis in NIU

### 4.1 Increased intestinal permeability and gut microbial translocation

Alterations in the gut microbiome may affect intestinal permeability and lead to leaky gut, which would allow the translocation of gut microbes and their products into the systemic circulation, thereby triggering innate and/or adaptive immune responses. Our study found that serum Zonulin levels in patients with BD and Fuchs syndrome were significantly higher than those in healthy controls (87). Zonulin is a physiological regulator released by intestinal epithelial cells after exposure to microorganisms or a gluten diet and can degrade intercellular tight junctions in the intestinal barrier (93). Moreover, early studies also reported increased intestinal permeability in BD patients without symptoms or signs of gastrointestinal disease (94). Patients with AU were also found to have chronic intestinal inflammation by ileocolonoscopy, which was associated with the recurrence rate of uveitis, but not with HLA-B27 status, sacroiliitis, or Nonsteroidal Anti-inflammatory drug (NSAID) intake (95). These findings were confirmed in B10.RIII EAU mice. The maximum changes in intestinal morphology (including ileal villus length, crypt depth, submucosal thickness, and muscular layer thickness) occurred prior to the peak of uveitis, which coincided with the peak expression of intestinal Zonula-occludens-1 (ZO-1), increased production of antimicrobial peptides (AMPs), and a reduction in cytokine production. These changes were reversed at the peak of uveitis. However, the increase of intestinal permeability and the difference of intestinal bacteria was paralleled to the course of uveitis, both reaching the peak on day 14 after immunization (13). Interestingly, Wang et al. also revealed intestinal barrier disruption in mice receiving feces from patients with active BD, as shown by decreased expression of three tight junction proteins (Claudin1, Claudin4, and Occludin) in colon tissues and a significant increase in LPS in serum (65). The above evidence suggests that increased intestinal permeability may be an early marker of uveitis and is closely related to the changes in the intestinal microbiome.

In LPS-induced uveitis (EIU) models, high-dose ^13^C-labeled propionate administered intraperitoneally can be detected in the ocular tissues. Unlabeled propionate was also detected in the eye, which may be produced by gut bacteria and enter the eye via the systemic circulation, or generated by ocular cells metabolizing pyruvate (96). These results suggest the existence of the gut-eye axis. The EIU model is an animal model resembling human AAU. Therefore, future studies are needed to further investigate whether orally ^13^C-labeled SCFA can translocate through the gut and be transferred to the eye in EAU mice (posterior uveitis model). Notably, the translocation of bacterial components or products, such as LPS and SCFA, has only been observed so far. No study has reported the translocation of intact bacteria in patients or mouse models of uveitis, which may be due to the existence of the intestinal vascular barrier (97) and blood-retinal barrier (BRB) (98), which limit the systemic spread of intact bacteria and reach the target organ, the eye.

The translocation of lymphocytes or other inflammatory cells from the gut to the eye is also considered one of the mechanisms involved in the pathogenesis of uveitis. The migration of gut-derived pathogen-associated molecular patterns (PAMPs) increases antigen exposure in the lamina propria, thereby promoting the differentiation of intestinal immune cell subsets. These immune cells may subsequently migrate to extra-intestinal target organs, and lower the threshold for extra-intestinal inflammation (99, 100). As previously described, cell motility *in vivo* can be detected using the photoconvertible fluorescent protein Kaede (101). Building on this methodology, Nakamura et al. demonstrated that lymphocyte migration from the gut to extra-intestine lymph nodes and the eye was enhanced in Kaede transgenic C57BL/6J EAU mice (66). Additionally, the research group also found that Th1 and Th17 cells in the mesenteric lymph nodes (MLN) of B10.RIII EAU mice increased on day 7 post-immunization, accompanied by a decrease in intestinal cytokine levels. This intestinal change may contribute to the migration of leukocytes from the gut to extra-intestine lymph nodes (13).

### 4.2 Molecular mimicry and bystander activation

Molecular mimicry may represent another mechanism by which the gut microbiome contributes to the pathogenesis of uveitis. The spontaneous uveitis mouse model (R161H) expresses specific T cell receptors (TCR) against IRBP peptides on B10.RIII mice background. Disease signs appear around weaning age, and all mice develop uveitis by 2 months of age in R161H mice. Unlike the induced model (EAU), in the spontaneous model, retina antigens are sequestered within the eye, while retina-specific T cells in the peripheral circulation must be activated before crossing BRB to initiate intraocular inflammation. Therefore, the question of where pathogenic T cells are activated has attracted attention. Activated Th17 cells have been detected in the intestinal lamina propria of R161H mice before the onset of ocular inflammation. In addition, R161H-Rbp3-/- mice do not develop uveitis due to the lack of target antigen. However, they have a high frequency of Th17 cells in the gut, similar to that of R161H-WT mice, and the transfer of activated T cells into naive wild type (WT) mice can induce uveitis. Moreover, *in vitro* studies have also found that R161H T cells are activated in an MHC class II-dependent manner by gut contents rich in bacteria (102). These observations all support gut microbiome as a trigger of uveitis. Nakamura et al. treated EAU mice with different antibiotics (metronidazole, vancomycin, neomycin, and ampicillin) to narrow down the gut bacteria species that influenced the severity of uveitis. Studies have shown that oral administration of metronidazole or vancomycin alone significantly reduces the clinical score of uveitis, accompanied by a reduction in the abundance of *Coprococcus*, *Dorea*, *Clostridium*, and *Lactobacillus*, which may be potential sources of mimic antigens (14). However, the other two antibiotics did not ameliorate uveitis. The team of Caspi identified a series of sequences homologous to IRBP_161-180_, which derived from the gut bacterium *Turicibacter*. However, these sequences failed to induce T cell proliferation from R161H mice and did not provoke disease when immunized in mice. The mimic antigens that induce disease may vary in different uveitis entities (103). Therefore, more studies are needed to identify microbiome peptides that have homologous sequences with retinal antigens (including IRBP and retinal S-antigen) and melanocyte antigens (target antigens of VKH disease, including tyrosinase and gp100).

The gut microbiome can also modulate immune responses via adjuvant effects. EAU (induced model) mice rely on active immunization by emulsifying the IRBP antigen in complete Freund’s adjuvant (CFA, such as heat-killed *Mycobacterium tuberculosis*). CFA strongly stimulates the innate immune response by activating antigen-presenting cells through pathogen recognition receptors, thereby presenting the IRBP antigen in this context and inducing autoreactive T cells (103). Fecal transplantation from patients with active BD did not induce ocular inflammation in naïve mice, but it affected the composition of the gut microbiome, the intestinal barrier, and T cell differentiation in the mesenteric lymph nodes (MLN) and spleen (65). However, EAU induction after fecal transplantation aggravated the development of uveitis (16, 65). They also found that *Stenotrophomonas*, which was significantly enriched in BD patients, encodes a microbial peptide SteTDR_9-17_ (YVQPGNTIL) that is homologous to IRBP_161-180_ (YLHPGNTIL). This peptide can stimulate PBMCs from BD patients or lymphocytes from EAU mice to produce IFN-γ and IL-17. However, similar to the antigen peptides identified by Caspi, SteTDR_9–17_ cannot induce uveitis directly in mice, but it can synergize with IRBP, amplifying the activated state in a bystander manner, thereby exacerbating EAU (104).

### 4.3 Blood-retinal barrier

BRB serves to sequester retinal antigens within the eye, thereby preventing their recognition by the peripheral immune system and inhibiting the entry of unactivated T cells into the retina, thus averting retinal inflammation (98, 102). However, in cases of gut microbiome imbalance, retina-specific T cells can become activated, entering the peripheral circulation and potentially inducing inflammation through the BRB (102). Notably, in EAU mice, an increase in BRB permeability was observed, as evidenced by widespread Evans blue staining leakage in retinal vessels (105, 106). Xiang et al. also reported a reduction in occludin levels, a key component contributing to BRB integrity, in the retina of EAU mice (106). Intriguingly, EAU mice treated with propionate or antibiotics exhibited decreased BRB permeability (14, 66, 107). These observations suggest a potential link between gut microbiome dysregulation and altered BRB permeability, implying that antibiotics or SCFAs may offer beneficial effects in preserving BRB integrity.

### 4.4 Immune responses

Alterations in the gut microbiota can also impact immune responses. Th17 cells are a crucial pathogenic subset in autoimmune uveitis. In R161H mice, broad-spectrum antibiotic treatment reduced intestinal Th17 cell activation and alleviated uveitis, while *in vitro* studies demonstrated that bacteria-rich intestinal extracts directly activated IRBP-specific T cells, highlighting the role of gut microbial antigens in driving autoreactive T cells (102). Similarly, germ-free (GF) mice exhibited decreased IFN-γ and IL-17 production and increased Tregs in ocular draining lymph nodes compared to conventionally housed EAU mice (108). Nakamura et al. further reported that, prior to the onset of uveitis, expansion of Th1 and Th17 cells in mesenteric lymph nodes (MLN), along with suppressed intestinal cytokines, may promote the migration of pathogenic lymphocytes from the gut to peripheral tissues and the eye (13, 66). Furthermore, colonization of *C. scindens* associated with secondary bile acid production was found to diminish the proportions of Th1 cells, Th17 cells, and DCs in the spleen of EAU mice, emphasizing the systemic immunomodulatory effects of specific gut microbes (73).

Besides adaptive immunity, gut microbiota dysbiosis also affects innate immune responses in NIU. For instance, fecal microbiota from patients with BD can induce neutrophil activation when transferred to mice, which contributes to the differentiation of pathogenic T cells (65). Microglia, the resident tissue-specific macrophages in the central nervous system, including the retina, are also influenced by gut microbiota. Activated microglia can disrupt the integrity of the BRB and recruit peripheral leukocytes (109, 110). In EAU mice, minocycline treatment inhibits microglial activation and reduces IL-1β production by microglia/macrophages (107). These findings collectively support the crucial role of gut microbiota in orchestrating both innate and adaptive immune responses contributing to the pathogenesis of NIU.

Overall, it is not contradictory that the gut microbiome may be the source of both antigens and adjuvants, making it a trigger and/or amplifying signal for uveitis-specific T cells. Cross-reactive antigens are not provided by a single species but are the result of the collective action of multiple microbes. The gut microbiome that provides mimic antigens remains to be identified. Based on these hypothesized mechanisms, several potential intervention strategies can be considered. Restoring intestinal barrier function may prevent microbial translocation and systemic immune activation, such as by modulating Zonulin signaling. Moreover, targeting gut microbiome dysbiosis through selective antibiotics, probiotics, or dietary interventions may help restore microbial balance and limit bacteria capable of producing mimic antigens or pro-inflammatory metabolites. These strategies may complement existing immunosuppressive therapies and offer novel microbiome-targeted approaches for NIU management.

## 5 Potential therapeutic strategies for NIU based on gut microbiome

**Figure 3:**
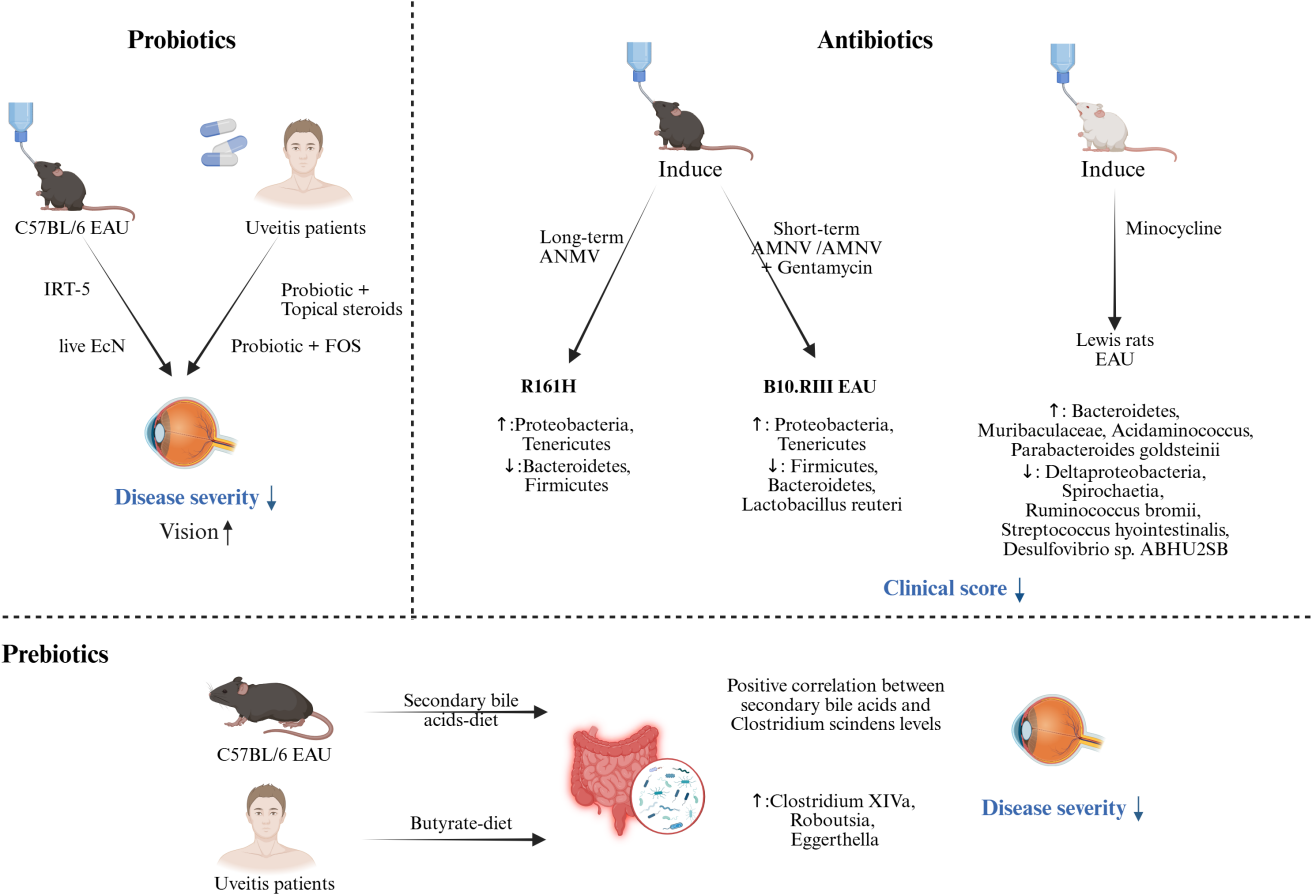
Probiotics, prebiotics, and antibiotics administration as a potentially beneficial strategy against uveitis. Probiotics. Preclinical studies through the EAU model have shown that probiotics used before or during immune induction can significantly reduce clinical scores. Clinical studies have found that combining conventional treatment (topical steroids) or prebiotics with probiotics can also significantly reduce disease severity and improve vision. Prebiotics. A significant positive correlation between secondary bile acid levels and Clostridium scindens was found in EAU mice. Clinical studies have highlighted changes in gut microbiome composition, with relative increases in Clostridium XIVa, Roboutsia, and Eggerthella, following supplementation with a butyrate-rich diet. Antibiotics. Preclinical studies have shown that long-term (oral administration of ANMV from maternal pregnancy to offspring after weaning) administration of ANMV in R161H mice significantly affected the gut microbiome composition (increased Proteobacteria, Tenericutes, and decreased Bacteroidetes, Firmicutes). Short-term administration of ANMV or ANMV+gentamicin (from one week before induction to the end of the experiment) also significantly affected the gut microbiome in B10.RIII induced EAU mice. (Created with BioRender.com) IRT-5 (Lactobacillus casei, Lactobacillus acidophilus, Lactobacillus reuteri, Bifidobacterium bifidum, Streptococcus thermophilus); fructo-oligosaccharide (FOS); Escherichia coli Nissle 1917 (EcN); a broad-spectrum antibiotic cocktail of ampicillin, metronidazole, neomycin and vancomycin (AMNV); Experimental autoimmune uveitis (EAU).

Currently, widely used systemic treatments for uveitis include corticosteroids, immunosuppressants (such as cyclosporine, methotrexate, mycophenolate mofetil), and biologics. However, these drugs have significant side effects and can greatly affect the composition of the gut microbiome. For example, mycophenolate mofetil (MMF) and methotrexate (MTX) treatment of EAU mice induced significant but distinct changes in intestinal bacterial composition (111). MMF may alleviate uveitis by expanding the Treg subsets in the MLN and the eye. In contrast, MTX significantly suppresses the Teff subsets in most tissues, maintaining the disease in a quiescent state, but the risk of relapse may be higher after drug withdrawal (111). Therefore, in this section, we will discuss the impact of probiotics, prebiotics, dietary modifications, antibiotics, and fecal microbiome transplantation (FMT) on immune responses (**Table 3**), gut microbiome, and disease activity (**Figure 3**).

**Table 3:** Preclinical studies on the immunology effects of treatment targeting the gut microbiome.

Animal model	Intervention	Treatment duration	Resulting immune responses	Country
Probiotics				
IRBP_1–20_ induced EAU, C57BL/6J (113)	IRT-5	Day 0 post-immunization until the end of experiment (3 weeks), daily treatment	↓: CD8^+^IL17^hi^ and CD8^+^IFNγ^hi^ cells in CLN.	Korea (2017)
IRBP_1–20_ induced EAU, C57BL/6J (115)	EcN or EcO	3 times per week. Four treatment schedules. Live or autoclaved bacteria.	↓: IRBP-specific IFN-γ^+^CD4^+^ and TNF-α^+^CD4^+^ T cells in ILN; ↓: iNOS^+^ macrophage (pro-inflammatory M1 macrophage) proportion in MLN.	Czech Republic (2020)
Prebiotics				
IRBP_1-20_/IRBP_651–670_ and IRBP _161–180_ induced EAU, C57BL/6J (66)	Prop, Buty, Acet	C57BL/6J EAU: 21 days pre-immunization until the end of experiment (21days + 4 weeks); B10.RIII EAU: 21 days pre-immunization until the end of experiment (21 days + 3 weeks)	↑: Treg cells by Prop in eyes/CLN at week 4 post-immunization; ↓: Th1 and Th17 by Prop in CLN/MLN at 1 week post-immunization; Inhibition of Teff cell migration between intestine and eye.	United States (2017)
IRBP_1–20_ induced EAU, C57BL/6J (67)	Buty	Day 7 to day 14 post-immunization (7 days)	↑: Treg; ↓: Teff and DC; (in draining lymph nodes and spleens)	China (2017)
IRBP_651–670_ induced EAU, C57BL/6J (73)	Secondary	Day 0 to day 14 post-immunization (14 days)	↓: Th1, Th17 cells, and CD11c MHC class II^+high^ DCs.	China (2021)
Diets				
IRBP _651–670_ induced EAU (68)	Pectin diet	5 weeks pre-immunization until the end of experiment (5 weeks + 1 or 2 weeks)	↑: Treg; ↓: Th1 and Th17 cells in various lymphoid tissues (including LPL, CLN, MLN, spleen and eye)	United States (2023)
Antibiotic				
R161H mice (spontaneous model), B10.RIII (102)	AMNV	Treatment was given to pregnant dams and continued after weaning.	↓: IL-17α^+^ T cell frequency in LPL of the small intestine and colon	United States (2015)
EAU, B10.RIII (102)	AMNV	Treatment was given to pregnant dams and continued after weaning.	No significant effect on disease severity improvement	
IRBP_161–180_ induced EAU, B10.RIII (14)	AMNV: Treatment with antibiotics alone or in combination; Oral or intraperitoneal antibiotics	One week pre-immunization until 3 weeks post-immunization (4 weeks)	One week post-immunization: ↑: Treg in LPL; ↓: Th1 and Th17 cells in extraintestinal lymphoid tissues; 3 to 4 weeks post-immunization: ↑: Treg in extraintestinal lymphoid tissues (including eyes)	United States (2016)
IRBP_1–20_ induced EAU, C57BL/6J (108)	Broad-spectrum antibiotics (metronidazole and ciprofloxacin)	One week pre-immunization or on the day of immunization until the end of experiment (1 week +35 days or 35 days)	No significant difference.	Australia (2016)
IRBP_161–180_ induced EAU, B10.RIII (36)	AMNV + Gentamycin	Short-term treatment: One week pre-immunization until the end of experiment; (1 + 2 weeks) Long-term treatment: Ten weeks pre-immunization until the end of experiment (10 + 2 weeks)	A gradual disappearance of the CD4+ and CD4+CD8+ subset of gut intraepithelial lymphocytes (IELs). L. reuteri correlated with the frequencies of CD4+CD8+ IELs.	United States (2023)
IRBP_161–180_ induced mice, Lewis rats (107)	Minocycline	a. Day 0 to day 14 post-immunization; b. Day 4 to day 18 post-immunization. (14 days)	Suppression of microglia activation and down-regulation of IL-1β level in retina. ↓: macrophage and Th17 cell infiltration, and expression of MHC II in the retina.	China (2020)

IRT-5 (Lactobacillus casei, Lactobacillus acidophilus, Lactobacillus reuteri, Bifidobacterium bifidum, Streptococcus thermophilus); Escherichia coli Nissle 1917 (EcN); E. coli O83:K24:H31 (EcO); mesenteric lymph node (MLN); cervical lymph node (CLN); inguinal lymph node (ILN); lamina propria Lymphocytes (LPL); acute anterior uveitis (AAU); Behcet disease (BD); sodium propionate (Prop); sodium butyrate (Buty); sodium acetate (Acet); Dendritic cell (DC); a broad-spectrum antibiotic cocktail of ampicillin, metronidazole, neomycin and vancomycin (AMNV).

### 5.1 The intervention of probiotics and prebiotics

Probiotics are living microorganisms that benefit host health when administered in sufficient amounts (112). After reducing the gut microbiome load with antibiotics in advance, IRT-5 probiotics (*Lactobacillus casei*, *Lactobacillus acidophilus*, *Lactobacillus reuteri*, *Bifidobacterium bifidum*, and *Streptococcus thermophilus*) supplementation on the day of immunization in EAU mice alleviated the severity of uveitis and significantly inhibited the expansion of CD8+T cells in cervical lymph nodes (CLN) (113). Antigen-specific CD8+ T cells can also induce autoimmunity in uveitis models, although CD4+ T cells are predominant (114). Additionally, administration of live *Escherichia coli* Nissle 1917 (EcN) only before or during disease induction significantly reduced clinical scores in EAU, indicating that the immunoregulatory effects of EcN preceded the development of EAU and influenced the initial antigen presentation and T cell (IRBP-specific CD4+ T cell) activation in the draining lymph nodes of immune sites (115). Despite the transient colonization of EcN in the gut, administration of EcN from 2 weeks before immunization to the day of immunization similarly prevented uveitis, suggesting that the beneficial effects of EcN are not limited to local effects on the gut. The protective effect of EcN may lie in the improvement of intestinal barrier function and the extension of anti-inflammatory regulation of intestinal mucosal immunity to innate and adaptive immunity, thus having a long-term impact on host health (115). A case report on AAU patients showed that local corticosteroids combined with probiotic supplements can alleviate inflammation and reduce the frequency of recurrences (116). Similarly, in a BD patient with AAU, synbiotics (a combination of probiotics and prebiotics) demonstrated similar therapeutic effects (117).

Prebiotics can selectively stimulate the growth and/or activity of one or a limited number of bacteria in the gut microbiome, thereby improving host health (118). Common prebiotics include galacto-oligosaccharides, fructans, and inulin, which can be metabolized by intestinal bacteria to produce metabolites such as SCFA, secondary bile acids, and folate. These metabolites further regulate the composition of the gut microbiome and interact with the immune cells of the host (119). As previously mentioned, supplementation with SCFAs, secondary bile acids, or diets that produce SCFAs can significantly reduce the severity of inflammation in uveitis.

Taken together, preclinical studies suggest that different probiotic strains may trigger distinct immune mechanisms to alleviate uveitis. However, large cohort randomized controlled trials are needed to validate their preventive and therapeutic efficacy in clinical settings. Although probiotics and prebiotics may transiently modulate immune responses and improve symptoms, their long-term effects remain controversial. Prolonged colonization of probiotics may disrupt gut microbiome structure, reduce beneficial commensals, and replace native microbes essential for immune balance (120, 121). Additionally, if intestinal barrier integrity is compromised, probiotics may translocate into the systemic circulation, leading to invasive infections (120). Notably, in a clinical trial of patients with severe acute pancreatitis, synbiotic prophylaxis containing *Bifidobacterium*, *Lactobacillus*, cornstarch, and maltodextrin was associated with a 2.5-fold increase in mortality and bowel ischemia compared to placebo (122). Therefore, while short-term use appears beneficial, the chronic application of these interventions should be cautiously evaluated, with more data needed to assess their safety and efficacy in NIU patients over extended periods.

### 5.2 The intervention of dietary modifications

Disease activity in patients with uveitis may also be related to the daily diet. It has been observed that a diet rich in vitamins and polyunsaturated fatty acids may alleviate disease progression by regulating immune responses (123). *In vitro* studies have found that linoleic acid (LA) can inhibit the antigen presentation function of DCs by reducing the expression of co-stimulatory molecules. Meanwhile, LA can also decrease the differentiation of Th cells in both humans and mice, and suppress the secretion of inflammatory cytokines by retinal pigment epithelial cells [110]. Nevertheless, LA has traditionally been regarded as a proinflammatory factor, and its downstream metabolite, arachidonic acid, is capable of converting into pro-inflammatory eicosanoids such as prostaglandin E2 (124). The level of LA was also significantly elevated in AU and BD patients (74, 87, 89, 91, 125). These different results suggest that the increase of LA in uveitis may be a feedback mechanism for the immune response suppression, which requires further investigation. Moreover, a double-blind randomized controlled trial found that combined vitamin C and E supplementation could improve visual acuity in AAU patients (126). Additionally, Li et al. found that caloric restriction increased Treg cells, altered the metabolic state of immune cells, and downregulated glycolysis-related genes by single-cell RNA sequencing. Further flow cytometry analysis confirmed that caloric restriction modulates the PI3K/AKT/c-Myc signaling axis and reduces granulocyte-macrophage colony-stimulating factor (GM-CSF) production in Th17 cells, thereby promoting CD4^+^ T cell balance (127). Similarly, Duan et al. demonstrated through single-cell RNA sequencing that a ketogenic diet (high fat, low carbohydrate) increased Treg cells, reduced Th17 cells, and restored the Th17/Treg balance, thereby alleviating EAU progression. The diet also partially reversed inflammatory responses in retinal immune cells, especially CD4^+^ T cells (128).

Overall, dietary interventions, such as fermentable fiber-enriched diets, ketogenic diets, or caloric restriction, have demonstrated promising immunomodulatory effects in preclinical uveitis models. These strategies may help maintain immune balance and prolong remission periods in NIU. However, long-term dietary intake also affects gut microbial structure and function (129). Cross-sectional studies have shown that long-term dietary intake, rather than short-term intake, is strongly associated with enterotype distribution, with high-protein and animal fat diets favoring Bacteroides-dominant enterotypes, while high-carbohydrate diets are linked to Prevotella-dominant enterotypes (129). Nevertheless, strict adherence to specific dietary patterns over prolonged periods may lead to nutritional deficiencies, metabolic disturbances. For example, although low-carbohydrate diets may promote weight loss and metabolic benefits (130), insufficient carbohydrate intake can reduce the abundance of butyrate-producing bacteria and fecal butyrate concentrations, potentially impairing gut and systemic immune homeostasis (131). Therefore, individualized dietary planning, coupled with close nutritional and clinical monitoring, is essential to ensure safety and effectiveness over extended periods.

### 5.3 The intervention of antibiotics

In several studies of uveitis models, raising mice under germ-free conditions or depleting the gut microbiome with antibiotics significantly reduced the severity of the disease (14, 36, 102, 107, 108). Notably, EAU models suggest that the beneficial effects of antibiotics in uveitis are largely dependent on early intervention, supporting their role as a prophylactic rather than a therapeutic agent. This preventive capacity appears to depend on several factors: 1) the time point of antibiotic administration, 2) the duration of antibiotic treatment, 3) the type of antibiotic used, and 4) the method of antibiotic administration. Heissigerova et al. found that metronidazole and ciprofloxacin treatment started on the day of EAU induction did not significantly affect the clinical score of uveitis, while intervention one week in advance alleviated the disease, suggesting that pre-existing gut microenvironment may affect EAU susceptibility (108). In a separate study, Seidler Stangova et al. demonstrated that metronidazole monotherapy administered two weeks before disease induction significantly attenuated EAU severity, further supporting its role as a prophylactic agent through early intervention (132). In R161H mice, oral administration of broad-spectrum antibiotics (ampicillin, metronidazole, neomycin, and vancomycin, AMNV) starting from the pregnancy of the dam, and continuing the treatment after weaning in the offspring, significantly delayed the development of uveitis. Meanwhile, the frequency of IL-17α+T cells and the cytokines IFN-γ and IL-22 in the lamina propria of the small intestine were decreased after AMNV intervention, but no expansion of Treg cells was observed. Under the same experimental conditions, EAU mice treated with AMNV were not significant improved (102). In contrast, Nakamura et al., also using AMNV treatment, showed significant improvement in uveitis when oral rather than intraperitoneal antibiotics were started one week before immunization. Moreover, this study also found that short-term antibiotic intervention could increase the frequency of Tregs in the intestinal lamina propria at one week post-immunization and increase Tregs in the extra-intestine lymphoid tissue at 3 or 4 weeks post-immunization, while Teff cells were reduced in the extra-intestine lymphoid tissue at the early stage of disease (14). Based on the different findings from two studies, Caspi and colleagues further investigated the impact of antibiotic treatment duration on EAU. The results showed that short-term antibiotic intervention could eliminate the microbiome stimulating the disease, thereby providing a protective effect. However, with prolonged antibiotic intervention, the protective microbiome was also gradually depleted and became resistant, along with secondary depletion of regulatory intestinal intraepithelial lymphocytes (IEL, which inhibit IRBP-specific T cell activation), and the protective effect was reversed. IELs are microbiome-dependent and are significantly reduced or absent in GF mice. After antibiotic intervention, the reduction of CD4+CD8+ IELs lagged behind the depletion of *Lactobacillus reuteri* (36). Nakamura et al. also found that metronidazole or vancomycin alone could also reduce inflammation and screened different kinds of protective microbes (14). In addition to regulating T cell differentiation, antibiotics can also suppress the activation of retinal microglia, thereby reducing the release of pro-inflammatory cytokines and the recruitment of inflammatory cells, ultimately inhibiting the onset and progression of uveitis (107). Under GF conditions, uveitis could be alleviated, but the disease still progressed after cohousing with SPF mice (102). Moreover, continuous antibiotic intervention led to dynamic and progressive changes in both the gut microbiome and immune cells, accompanied by a gradual increase in disease scores (36). These results suggest that microbial exposure is not the only trigger of disease development.

Generally, preclinical studies suggest that targeted short-term antibiotic interventions may ameliorate uveitis by reshaping the gut microbiome and modulating systemic immune responses. However, prolonged or repeated antibiotic exposure poses significant risks, including depletion of protective commensals, emergence of resistant strains, disruption of immune homeostasis, and loss of microbiome resilience (133, 134). Additionally, such interventions may increase susceptibility to opportunistic infections and exacerbate systemic immune dysregulation in susceptible individuals (134). Therefore, more targeted narrow-spectrum antibiotics are developed and used, and combined probiotics are considered to support microbiome recovery.

### 5.4 Fecal microbiome transplantation

FMT is the transplantation of fecal microbiome from healthy donors into the gastrointestinal tract of patients to compete for the niche with pathogenic strains, thereby reshaping the intestinal microbiome (135). FMT has become a recommended treatment for recurrent *Clostridioides difficile* infection (136, 137), as it helps restore gut diversity, improve metabolic function, and modulate the immune system (138). Due to its significant therapeutic potential, FMT has also been gradually applied to intervene in autoimmune diseases (139–141)]. However, FMT protocols involving different doses and routes of administration can cause a variety of clinical responses and lack reproducibility (142). In addition to bacteria, the fecal material used in FMT also contains viruses, fungi, archaea, and various metabolites. While more precise approaches such as fecal virome transplantation (FVT) and washed microbiota transplantation (WMT) have been developed to improve safety by minimizing unwanted components (143, 144), these methods are still experimental, and their long-term impacts remain unclear.

Notably, FMT has been associated with transmission of multidrug-resistant organisms, fungi, and viruses in certain cases (145). Moreover, persistent alterations of the gut microbiome induced by FMT may disrupt immune homeostasis, potentially leading to disease flares in patients with underlying immune disorders, such as IBD (146, 147). To mitigate these risks, next-generation FMT strategies are focusing on the transplantation of more defined microbial components, including WMT, bacterial spore formulations, and FVT, which aim to enrich beneficial taxa while minimizing undesirable elements and reducing the risk of adverse events (142). Additionally, advances in multi-omics technologies and refined donor-recipient matching models are expected to enhance donor screening precision and optimize FMT efficacy and safety in the future (142).

## 6 Conclusion

Despite the accumulating evidence linking gut microbiome dysbiosis to NIU pathogenesis, the translation of these findings into clinical practice remains challenging. Current insights propose that targeting the gut-eye axis through interventions such as modulating gut microbiome composition and key metabolites, as well as restoring intestinal barrier function, may offer novel adjunctive strategies alongside conventional immunosuppressive therapies. Specifically, strategies including dietary interventions, probiotics, prebiotics, or defined microbial communities warrant further exploration in NIU. However, future research should also focus on the following priorities to facilitate clinical translation. First, the specific pathogenic or protective microbial species, microbial-derived metabolites, and mimic antigens relevant to different NIU subtypes remain to be identified. Second, establishing causality between gut microbiome alterations and NIU onset, activity, or prognosis requires longitudinal clinical studies, mechanistic investigations using humanized animal models. Third, individualized interventions based on microbiome profiles and the immune status of patients should be developed. Addressing these priorities will help bridge the current gap between basic research and clinical translation and may open new avenues for precision medicine strategies in NIU management.
